# Photo Quiz: An uncommon culprit in a young woman with headaches—a numb face and a fungal trace

**DOI:** 10.1128/jcm.01127-25

**Published:** 2025-12-17

**Authors:** Tsung-Yu Tsai, Tzu-Ching Su, Pei-Lun Sun, Pei-Wen Wu, Tzong-Yow Wu, Liang-En Hwang, Aristine Cheng, Kuan-Yin Lin, Yee-Chun Chen

**Affiliations:** 1 Department of Internal Medicine, National Taiwan University Hospital and College of Medicine 568619, Taipei, Taiwan; 2 Department of Oncology, National Taiwan University Hospital 172673, Taipei, Taiwan; 3 Department of Dermatology, Chang Gung Memorial Hospital, Linkou Branch 38014, Taoyuan, Taiwan; 4 College of Medicine, Chang Gung University 71589, Taoyuan, Taiwan; 5 National Institute of Infectious Diseases and Vaccinology, National Health Research Institutes 50115, Miaoli County, Taiwan; 6 Division of Rhinology, Department of Otolaryngology, Chang Gung Memorial Hospital 125574, Taoyuan, Taiwan; 7 Department of Internal Medicine, National Taiwan University Hospital Yunlin Branch, Yunlin, Taiwan; 8 Department of Internal Medicine, National Taiwan University Hospital Biomedical Park Hospital 569163, Hsinchu, Taiwan; Mayo Clinic Minnesota, Rochester, Minnesota, USA

## PHOTO QUIZ 

A 35-year-old woman presented with right facial numbness and progressive occipital headaches for 1 month. Despite being negative for rheumatologic and antiphospholipid antibodies, she received belimumab, rituximab, methylprednisolone, and adalimumab for infertility over the past 2 years. Her symptoms began with right facial swelling and numbness in the maxillary region, nasal stuffiness, and upper toothache, followed by worsening bilateral occipital headaches 3 weeks later. She reported nearby construction and mountain camping, but denied animal or plant exposures.

Otolaryngologic evaluation revealed bilateral inferior turbinate congestion, nasal septal deviation, and an obliterated right osteomeatal complex with mucoid-purulent discharge filling the right superior meatus. Laboratory investigations showed leukocytosis (13,600 /µL; reference 3,250–9,160) and elevated C-reactive protein (2.27 mg/dL; reference <1). Computed tomography confirmed bilateral sinusitis, with extensive mucosal thickening. Functional endoscopic sinus surgery (FESS) was performed for debridement and culture sampling. Two weeks later, she developed fevers, worsening right maxillary pain, and headache unresponsive to piperacillin-tazobactam and teicoplanin, despite sinus cultures growing *Serratia marcescens*, *Cutibacterium acnes*, methicillin-resistant *Staphylococcus epidermidis*, and an unidentified mold.

**Fig 1:**
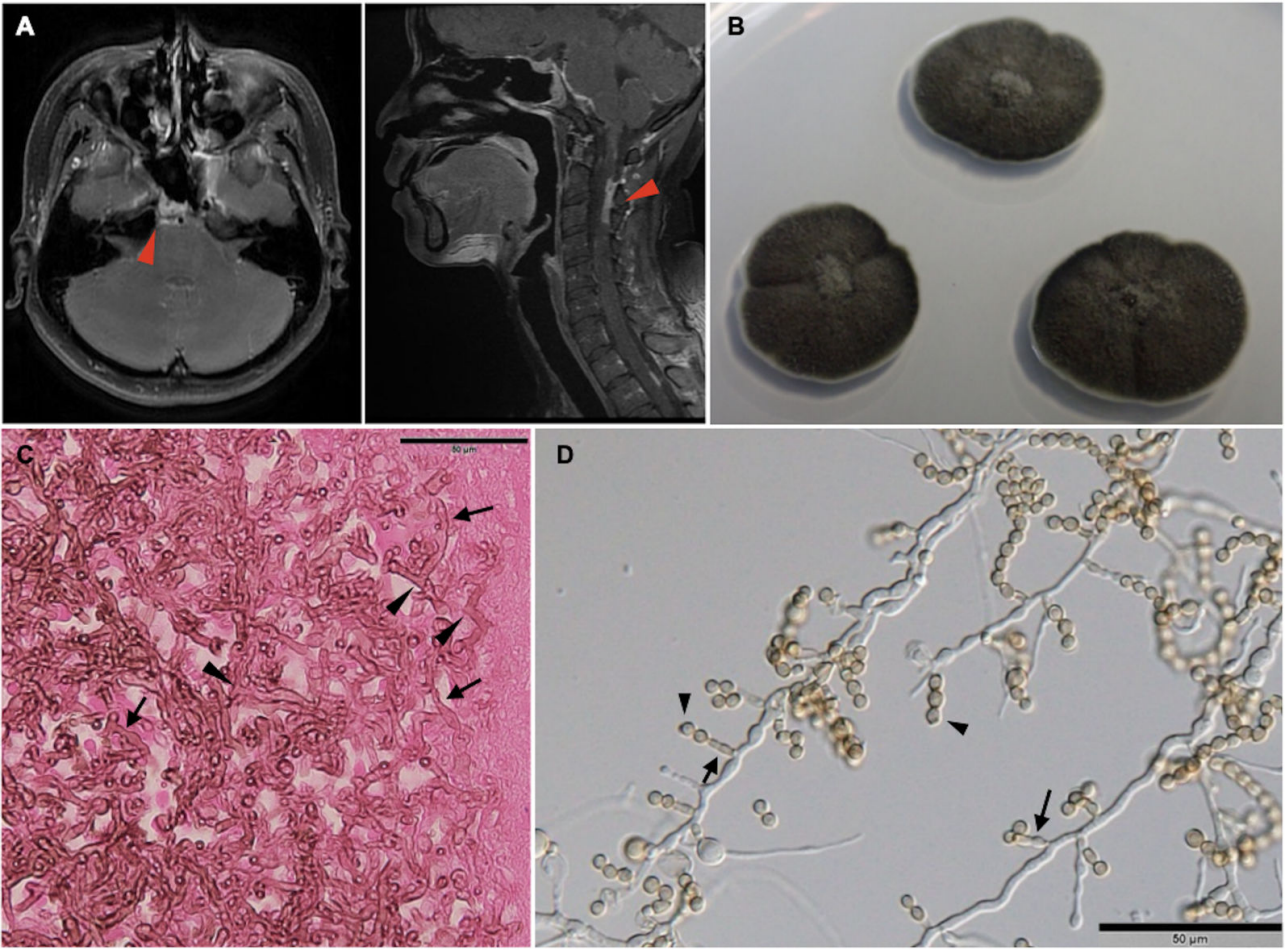
(**A**) Magnetic resonance imaging disclosed leptomeningeal enhancement in the peripontine and cervical spine areas (triangle). (**B**) Fungal culture on potato dextrose agar grew dark olive-green, round, velvety mold colonies with shallow radiating furrows. (**C**) Histopathology examination of the sinus specimen reported fungal hyphae with tissue invasion, including septate (arrow) and brown-pigmented branching hyphae (triangle) on hematoxylin and eosin stain. (**D**) Slide culture (unstained, scale bar = 50 µm) demonstrated vegetative hyphae along with annellides (arrow) and chains of round conidia in basipetal succession, displaying brown pigmentation (triangle).

Magnetic resonance imaging (MRI) disclosed widespread sinus opacification and leptomeningeal enhancement in the peripontine and cervical spine areas (Fig. 1A). Lumbar puncture revealed an opening pressure of 10 cmH_2_O (reference 9–18). Cerebrospinal fluid analysis indicated 525 WBCs/μL (reference 0–5), 49% lymphocytes, glucose 19 mg/dL (reference 40–70), and total protein 189 mg/dL (reference 15–45). Histopathology of the sinus specimen confirmed fungal hyphae with tissue invasion after repeated FESS sampling. Culture of the specimen on potato dextrose agar at 25°C grew dark olive-green, velvety colonies with shallow radiating furrows and a dark gray reverse, reaching approximately 18 mm in diameter by 3 weeks. Microscopically, the molds showed vegetative hyphae with annellides and chains of round conidia, displaying brown pigmentation (Fig. 1B through D). Liposomal amphotericin B (L-AmB) (5 mg/kg/day) was initiated.

What is your diagnosis?

## ANSWER TO PHOTO QUIZ

She was diagnosed with invasive *Microascus* infection, involving sinuses and leptomeninges. Numbness and headache were attributed to leptomeningeal inflammation. *Microascus* spp. are the teleomorphs of *Scopulariopsis* species and form olive-green colonies with white margins and a pale-brown reverse (1). Microscopic examination of the cultured isolate revealed filamentous, septate hyphae with chains of round-to-oval conidia produced from annellides in basipetal succession, with the youngest conidia at the base and the oldest at the apex. Identification of *Microascus* spp. was confirmed by Sanger sequencing (Supplement).

*Microascus* spp. have variable susceptibility to antifungal agents, with limited activity of AmB and most triazoles (minimum inhibitory concentration range, 1 to >16 µg/mL) without established breakpoints (2). Therefore, combined therapy with high-dose L-AmB (10 mg/kg/day) and voriconazole (4–5 mg/kg twice daily) was subsequently administered, based on antifungal susceptibility tests per Clinical and Laboratory Standards Institute standards (AmB: 4 µg/mL; voriconazole: 4 µg/mL; posaconazole: 1 µg/mL; terbinafine: 2 µg/mL; anidulafungin: >16 µg/mL; and micafungin: >16 µg/mL) and better central nervous system (CNS) penetration of voriconazole compared with other azoles. This regimen stabilized symptoms, but tapering L-AmB caused deterioration and obstructive hydrocephalus, requiring ventriculoperitoneal shunting. After 12 months of therapy, MRI demonstrated partial remission. Voriconazole-associated periostitis prompted switching to isavuconazole (200 mg/day). L-AmB was tapered and discontinued after 16 months, with sustained remission on MRI under isavuconazole monotherapy.

With compromised immunity, the patient may have acquired *Microascus* spp. through mountain camping, as these fungi inhabit soil and plants (3). Most studies referenced *Scopulariopsis* spp., with *S. brevicaulis* the most common cause of cutaneous, pulmonary, sinus, endocardial, and disseminated infections (3). Only six adult cerebral *Scopulariopsis/Microascus* cases were reported; most underwent surgery and received AmB and triazoles, but none achieved radiologic remission and survival (4–8). Given resistance, drug toxicity, and poor CNS penetration, management requires tailored antifungal therapy and surgery (9).

## SUPPLEMENTAL MATERIAL

Supplemental material jcm.01127-25-s0001.docx Methods for Sanger sequencing

ASM does not own the copyrights to Supplemental Material that may be linked to, or accessed through, an article. The authors have granted ASM a non-exclusive, world-wide license to publish the Supplemental Material files. Please contact the corresponding author directly for reuse.
