# Supplementary material for: Photo Quiz: An uncommon culprit in a young woman with headaches—a numb face and a fungal trace
Source: J Clin Microbiol. 2025 Dec 17;63(12):e01127-25. doi: 10.1128/jcm.01127-25 (PMC12710301; doi:10.1128/jcm.01127-25)
Supplement: Supplemental material — Methods for Sanger sequencing [file jcm.01127-25-s0001.docx]

**Supplementary Material**

Supplement to:

**An Uncommon Culprit in a Young Woman with Headaches – A Numb Face and a Fungal Trace**

Tsung-Yu Tsai, Tzu-Ching Su, Pei-Lun Sun, Pei-Wen Wu, Tzong-Yow Wu, Liang-En Hwang, Aristine Cheng, Kuan-Yin Lin, Yee-Chun Chen

**Methods for Sanger sequencing**

The partial 18S and 28S rRNA genes, along with the full-length ITS1-5.8S-28S region of ribosomal rDNA, were amplified using the primer pairs ITS5 and ITS4. The PCR product was confirmed by gel electrophoresis and sequenced using Sanger sequencing. Comparison with sequences in GenBank revealed a single best match: *Microascus* sp. ES-2025a (isolate URM 9260) with 99% similarity (532/534 nucleotides). URM 9260 was submitted by Silva et al. in May 2025 and deposited under the title “A polyphasic approach reveals *Microascus luzeae* (*Microascales, Microascaceae*), a new species from bats living in a cave in Brazil.” However, the publication is currently unavailable, and the name has not been formally listed in MycoBank. Consequently, its legitimacy remains unconfirmed, and the morphological descriptions of this potentially new species are unavailable for comparison. Therefore, we identified this isolate only to the genus level, which represents the most appropriate classification based on the data currently available.
